# Supplementary material for: Predictors associated with Clavien–Dindo complications in lung cancer surgery: A retrospective cohort study
Source: PLoS One. 2024 Dec 31;19(12):e0316214. doi: 10.1371/journal.pone.0316214 (PMC11687775; doi:10.1371/journal.pone.0316214)
Supplement: Attachment — Submitted filename: Responses to Reviewers.docx [file pone.0316214.s003.docx]

Responses to Reviewers

Reviewer #1: Dr. Saetang and colleagues reported an interesting paper regarding postoperative complications and possible associated risk factors.
The article is of possible interest, but I have some comments.
1. most of the findings of the paper are somewhat already known. Authors should focus on what is brought as clinical new message by their own paper.

Response: Thank you for your suggestion. We agree that most of the findings of the paper are already known, however our findings highlight the development of risk assessment tool tailored to older lung cancer patients refining surgical decision making and postoperative care. We revised in Introduction (line 64-65), and conclusion (line 241-244.)

2. Some words should be revised (e.g. in table: "mode of surgery" should be changed with "extent of surgery"; "Operations" with "surgical technique")

Response: Thank you for your opinion. We have revised the terms in Table 2 and Table 4 to replace "mode of surgery" with "extent of surgery" and "operations" with "surgical technique" for improved clarity and accuracy.

3. In the Material and methods, definition of Clavien-Dindo complications could be put in a table rather then written in the text

Response: Thank you for your suggestion. We have moved the Clavien-Dindo classification definitions to a table in the Material and Methods section (line 82).

4. Results of complications according to the surgical technique (minimally invasive/open) or extent of surgical resection (segmentectomy/lobectomy/pneumonectomy/extended resection) should be reported in the text with more details and not only in the tables.

Response: We have already reported complications as Clavien-Dindo classification according to the extent of surgical resection in the Intraoperative and Postoperative Conditions section (line 113).

5. Moreover, I would try to perform a sub analysis of "best patients" (e.g. VATS, Lobectomy or sublobar resections, no conversion) and try to see if some of the other risk factors remain significant in this subgroup.

Response: Thank you for this insightful suggestion. We attempted a sub-analysis of patients who underwent segmentectomy or wedge resections; however, the sample size for these procedures was too small to perform meaningful statistical analysis. Consequently, no additional significant risk factors could be identified within this subgroup. We acknowledge this limitation and have noted it in the manuscript.

6. Another important factors are intraoperative complications, like severe bleeding. This should be taken into account in data analysis.

Response: We have already included blood loss and severe bleeding in **Table 4** as part of the analysis. However, we found that blood loss was not significantly associated with Clavien-Dindo classification ≥ II in this study.

7. the ROC analysis I think it is unnecessary.

Response: Thank you for your recommendation. We agree with your suggestion and have removed the ROC analysis from the manuscript as it was deemed unnecessary.

Reviewer #2: Good statistical analysis but I suggest to define elderly patient and divide younger from older patients in order to obtain more correct results. Obiouvsly elderly patients have much more postoperative complications.

Response; Thank you for your suggestion. We defined elderly patients as those aged ≥60 years and performed a subgroup analysis comparing patients aged 60–69 years and those aged ≥70 years. The analysis revealed that patients aged ≥70 years were more likely to have Clavien-Dindo grade ≥ II complications in the univariate model (OR: 1.92, 95% CI: 1.02–3.63, p = 0.045). However, after adjusting for other risk factors in the multivariable model, this association was not statistically significant (OR: 2.03, 95% CI: 0.95–4.36, p = 0.068). These findings suggest that age alone may not be an independent predictor of severe complications when other factors are considered. The revised analysis has been incorporated into **Table 1,4**  and discussed in the **Results (line** 138**)** and **Discussion** sections (lines 168).
